# Ethics and Values in Design: A Structured Review and Theoretical Critique

**DOI:** 10.1007/s11948-021-00329-2

**Published:** 2021-08-19

**Authors:** Joseph Donia, James. A. Shaw

**Affiliations:** 1 Institute of Health Policy, Management and Evaluation, Dalla Lana School of Public Health, University of Toronto, Toronto, ON Canada; 2 Joint Centre for Bioethics, Dalla Lana School of Public Health, University of Toronto, Toronto, ON Canada; 3 Women’s College Hospital, Toronto, ON Canada

**Keywords:** Design ethics, Values in design, Designer agency, Design strategy

## Abstract

A variety of approaches have appeared in academic literature and in design practice representing “ethics-first” methods. These approaches typically focus on clarifying the normative dimensions of design, or outlining strategies for explicitly incorporating values into design. While this body of literature has developed considerably over the last 20 years, two themes central to the endeavour of ethics and values in design (E + VID) have yet to be systematically discussed in relation to each other: (a) designer agency, and (b) the strength of normative claims informing the design process. To address this gap, we undertook a structured review of leading E + VID approaches and critiques, and classified them according to their positions on normative strength, and views regarding designer agency. We identified 18 distinct approaches and 13 critiques that met the inclusion criteria for our review. Included papers were distributed across the spectrum of views regarding normative strength, and we found that no approaches and only one critique represented a view characteristic of “low” designer agency. We suggest that the absence of “low” designer agency approaches results in the neglect of crucial influences on design as targets of intervention by designers. We conclude with suggestions for future research that might illuminate strategies to achieve ethical design in information mature societies, and argue that without attending to the tensions raised by balancing normatively “strong” visions of the future with limitations imposed on designer agency in corporate-driven design settings, “meaningful” ethical design will continue to encounter challenges in practice.

## Introduction

Technologies mediate our relationship to the world, and in doing so raise important moral questions (Winner, 1980). The continued expansion of technological capabilities through advances in artificial intelligence (AI) and Internet-of-Things (IoT) illustrate the urgency of addressing these moral questions. These technologies not only shape everyday human practices and engagement in life projects (Verbeek, 2006), but have rapidly re-created new social and economic orders that characterize the globalized world (Couldry & Mejias, 2019; Zuboff, 2019). The speed at which these changes have occurred and the lack of adequate response at the level of either national or supra-national policy has inspired renewed interest in the literature on ethics and values in design (E + VID).

While the literature on E + VID has developed considerably over the last 20 years (Davis & Nathan, 2015; Shilton, 2018b), we suggest that two themes central to the endeavour of E + VID demand further attention. The first theme is “designer agency”, referring to the conceptualization of the nature and extent of free human action engaged in by designers as they contribute to the design process. Whether designers are viewed as acting as a matter of their individual creativity, and the extent to which such creativity is understood to be both constituted and constrained by social relationships and structures, is consequential for the utility of E + VID. The second theme we address is “normative strength”, referring to the strength of moral claims or morally charged endpoints that are taken to motivate the design process. Although many scholars acknowledge that design is inherently normative (Feng & Feenberg, 2008; Verbeek, 2006), we suggest that stronger attention to the particular moral positions selected to frame a given design project would substantially deepen the relevance and impact of E + VID approaches. The purpose of this paper is to engage with these themes through a review of leading E + VID approaches and critiques according to the assumptions they make about designer agency and normative strength.

In doing so, our paper advocates a view of design that more explicitly acknowledges the broader ecosystem of influences on the designer’s ability to make intentional choices, and that more clearly states the normative positions informing those choices. Attending to the manner in which these ecosystems enable or constrain design can inform strategies for E + VID that go beyond the proximate issue of individual designer responsibility, to the institutional arrangements that have made the particular design project and its unique configuration of people and ideas possible. Without attending to these tensions, we argue that “meaningful” ethical design will continue to encounter both conceptual and practical challenges.

To provide context for our results and discussion, in “Background” we provide a brief introduction to E + VID, and introduce the theoretical background for our focus on designer agency and normative strength. We present our methods and results in “Methods” and “Results” respectively. To provide a finer analytic point on our findings, in “A Relational View on Designer Agency” we briefly summarize two contrasting views on agency that have been widely taken up in the literature: a relational approach to human agency (Emirbayer & Mische, 1998), and a network form of agency (Latour, 1999, 2005). We then conclude in “Future Directions and Conclusion” with suggestions for future research that may illuminate strategies to achieve ethical design in information mature societies.

## Background

There are a variety of approaches to considering ethics or values in the design of new technologies. While the politicization of design is most often associated with the Scandinavian participatory tradition exemplified by the UTOPIA project (e.g. Ehn et al., 1981), simultaneous developments in areas such as computer ethics (Moor, 1985) and science and technology studies (Winner, 1980) were also beginning to investigate the relationships between society, technology, and design. In 2002, Friedman, Kahn, and Borning drew explicit attention to the role of *values* in design, and a number of new frameworks have since built on these diverse traditions. While E + VID approaches vary considerably, they typically feature at least one of the following aims: (1) clarifying the normative dimensions of design, or (2) outlining strategies for explicitly incorporating values into design (Bardzell, 2010; Shilton, 2018b; van Wynsberghe & Robbins, 2014).

JafariNaimi et al. (2015) have labelled this general approach the identify/apply logic. This logic follows the assumption that many of the problems associated with new technologies arise from lack of engagement with values in design, and that we need better processes for scrutinizing values so that we may apply the “right ones” in practice. A second and more deeply implicit assumption is that designers do indeed have the capacity to critically reflect on the values being built into a given technology, and the ability to modify the role of values in design in order to bring about a more ethically designed product. However, within the identify/apply tradition, debate has primarily centred around whether values ought to be uncovered discursively, or descriptively through empirical research in use-contexts (JafariNaimi et al., 2015; Le Dantec et al., 2009).

Interest in these approaches has also been accompanied by questions about the appropriateness and feasibility of design as a site of ethical reflection and analysis. Aside from concerns about the enforceability of ethical design, scholars point to ethics washing, or the signalling of ethical concern without corresponding action (Bietti, 2020; Floridi, 2019; Wagner, 2018). Recent reviews of E + VID have also emphasized the importance of attending to questions about a designer’s ability to influence the moral trajectory of an artifact (Shilton, 2018b). If E + VID is to realize its aims of a more just, democratic, sustainable, or otherwise “good” society, engagement with questions of designer agency, and the normative strength of design approaches, will be essential.

### Designer Agency and Normative Strength

Central to the identify/apply logic of approaches to incorporating ethics and values into design processes are assumptions about the capabilities of designers. Specifically, this approach assumes that designers are capable of understanding the complex phenomenon of values, clearly delineating their relevance for a design activity, and then modifying design processes to reflect those values. These assumptions imply a particular understanding of designer agency, such that the designer is a morally motivated actor who is relatively unencumbered by the influence of social structures, perverse incentives, or limited social/cognitive capabilities (Feng & Feenberg, 2008). Stating these assumptions in this way brings to our attention the issue of how human agency is understood in the context of design, and what implications such understandings have for the normative relevance of approaches to E + VID.

The topic of the agency of designers has been addressed explicitly only rarely in E + VID literature (Feng & Feenberg, 2008; Woodhouse & Patton, 2004), which informs our motivation to complete the review reported in this paper. Debate about the nature of human agency has demanded a great deal of attention in the humanities and social sciences over the past centuries, and is an immensely complex topic (Emirbayer & Mische, 1998). In this paper we have the relatively modest aim of describing the assumptions embedded in E + VID literature regarding the extent to which designers are free to conceptualize values clearly, make sound decisions about their relevance to design, and act in ways that explicitly incorporate such values into design processes. We introduce the theoretical challenge of reconciling developments in social theory and philosophy that illustrate the heavy constraints on human agency with the necessary emphasis on creativity in design theory and practice (Selbst et al., 2019). However, we do not pretend to resolve this challenge in our paper, and instead invite the design scholarship community to engage with this challenge more deeply.

To set the context for our review, we do however outline some dimensions of the literature on human agency and clarify its links with the normative elements of design. Documented positions on the nature of the self and free human action in Euro-North American thought were for centuries dominated by the belief system of a Judeo-Christian religious worldview (Emirbayer & Mische, 1998; Merleau-Ponty, 1962; Taylor, 1992). Such a worldview includes the Enlightenment assumption that the human self is a contained entity free to make independent decisions, including an inanimate element (the soul in religious terms) that animates the material body (Rorty, 2009). Although this view was challenged in philosophical dialogue during the nineteenth century (James, 1896; Nietzsche & Zimmern, 1997), it was not until the twentieth century that this version of the human self became subject to sustained and direct critique. Philosopher Paul Ricoeur referred to three “masters of suspicion” in his work, arguing for the significance of Karl Marx, Friedrich Nietzsche, and Sigmund Freud as building the foundation for contemporary critiques of this dominant Judeo-Christian view of a free human agent (Ricoeur, 2008). Throughout the remainder of the twentieth century and into the twenty-first century, scholars from across the humanities and social sciences have been engaging deeply with assumptions about the human self and conceptualizations of agency that can more adequately represent the theoretical advancements of the past several decades. These theoretical advances will be addressed in more detail in the discussion section, and include a range of approaches to conceptualizing the human, creative action, and its role in the world (Bourdieu, 1977; Braidotti, 2013; Latour, 2005).

The implications of this history and the persistence of out-dated assumptions about the nature of human agency for E + VID can be stated quite clearly: If designers believe their work to be driven by the creative agency of individuals unencumbered by the substantial influence of the social and material world in the constitution of thought and action, they will overestimate their control over design processes and the ease with which particular normative positions can be advanced through design (Feng & Feenberg, 2008; Woodhouse & Patton, 2004). However, if designers and design scholars acknowledge theoretical insights about the nature of human agency, and recognize the co-constitution of thought and action by the individual and their social and material circumstances, they would pose new questions about the nature of incorporating ethics and values into design. These new questions would include a commitment to better understand the real sources of values and their related normative positions, whether those normative positions will actually contribute to a better future, and for whom that better future is envisioned.

The context presented here introduces the theoretical challenge that is central to our review of E + VID literature. Design is fundamentally viewed as a creative activity, and designers are celebrated for their ingenuity and individual skill. However, virtually all recent theory and evidence on human agency suggests that a more sophisticated understanding of design practice would more deeply acknowledge the ways in which designers’ vision and actions are constituted by influences outside of themselves.

To state the connections between the two areas of focus in our review more clearly, this point about designer agency is linked with the question of the normative strength of E + VID approaches in important ways. If designers were to acknowledge a more limited view of human agency, deeper thought would be put into the question of whether meaningful normative positions are actually being carried forward in E + VID, and how. Our review illustrates that the community of design scholarship and practice ought to consider the likelihood that limits to designer agency are deeply linked with limits in the normative strength and therefore moral significance of approaches to E + VID. We engage with this point more fully in the discussion section, and now turn to introduce the methods of our review.

## Methods

Our approach in this project was framed by a highly specific series of three objectives: (1) to identify a sample of leading approaches to E + VID that have been proposed and discussed in the literature, (2) to document the assumptions embedded in those approaches with respect to normative strength and designer agency, and (3) to categorize and describe the most notable critiques of those approaches related to normative strength and designer agency that have appeared since their publication. The objective was not to complete a thorough conceptual analysis of every E + VID approach, but rather to identify a group of generative publications and to produce statements of their positions regarding normativity and agency. In this way, our approach to identifying relevant publications was informed by purposive sampling in qualitative methods (Coyne, 1997), wherein researchers identify the characteristics of the data source that best match the specific objectives of their project. This approach has been described for its application to literature reviews as well (Suri, 2011).

We began with an informal scan of the literature based on known sources. This process uncovered several leading E + VID frameworks that were used as a starting point for additional searches through a process of systematic double-sided snowballing—or backwards and forwards reference checking and citation tracking (Contandriopoulos et al., 2010). The advantage of this approach over more formal database keyword searches is its ability to identify sources in heterogeneous bodies of literature spread across various disciplines and research traditions, where those traditions use different terms for similar concepts. Additional search strategies included authorship searches, reviews of existing E + VID syntheses, and keyword searches on Google Scholar.

Approaches selected for inclusion in our initial list were expected to meet the following 3 criteria: (1) were focused on the design process itself rather than ex post technology assessment; (2) were concerned with ethics or values in the design process; and (3) were highly cited, or otherwise recognized by peers as generative. Highly cited was defined as approximately five or more citations per year since publication. Papers outlining “sub-approaches”, defined as those that expanded upon or critiqued but did not substantially modify other existing approaches, were not included in the review. For example, a substantial overview of such approaches centring on value-sensitive design can be found in Davis and Nathan (2015). One exception in our review was Critical Technical Practice (Agre, 1997; Boehner et al., 2005) which includes both the initial generative contribution by Agre (1997), as well its more explicit orientation to values in design by Boehner et al. (2005).

Based on title and abstract screening, an initial sample of 22 papers were identified, each corresponding to a distinct approach to design ethics. Full-text screening was then performed, resulting in a final sample of 18 genuinely original frameworks that excluded sub-approaches as previously outlined. An extraction process summarized the frameworks’ descriptive and normative elements, which are outlined in the results section that follows.

Normative orientation was classified as strong, moderate, or weak. Although it could be argued that all design is normative in some respect, we used the term more narrowly to indicate *deliberate efforts to structure design processes and its outcomes to achieve a social goal* (Woodhouse & Patton, 2004). A strong normative orientation was therefore defined as explicit identification of *particular* values or normative ethical theories that ought to be mobilized to achieve a social goal. Normatively “moderate” approaches were those that gave primacy to a particular method or process for uncovering values (e.g. user involvement), but did not suggest that values or normative ethical theories ought to be mobilized to achieve a specific social goal. Normatively weak approaches did not make strict suggestions regarding which methods, values, or normative ethical theories ought to be brought to the design process, but may have offered a “menu” of values, theories, or approaches to consider depending on the design context.

Drawing on work by Woodhouse and Patton (2004) and Feng and Feenberg (2008), views on agency were classified as high, moderate, or low. “High” agency approaches framed design as primarily a technical task, occurring through negotiations with different actors and led by a designer or designers. “Moderate” approaches viewed design as a political task, where different social groups and their strategies affect the directionality of design. Finally, “low” agency approaches locate power at the macro-level, with culture substantially influencing the work of designers, or the design process and its priorities.

Following the description of generative approaches to E + VID, we purposefully examined contributions to the literature that have critiqued those approaches in specific ways. The purpose of this second literature search was to identify the central themes in literature that have critiqued E + VID in ways that directly relate to the two primary foci of our review (normative strength and designer agency). Again, the purpose was not to summarize every contribution to the literature in response to the generative approaches summarized in the first search, but rather to map the central themes that represent the ways in which scholars have critiqued E + VID literature from the perspective of normativity and agency.

The search process for critiques consisted of reference checking and citation tracking of all papers identified in the first search. Additional search strategies included authorship searches, reviews or syntheses of E + VID approaches, and keyword searches on Google Scholar. Papers were expected to meet the following inclusion criteria: (1) commented on or critiqued the E + VID literature; and/or (2) described an advancement in the E + VID literature. Title and abstract screening resulted in a preliminary sample of 55 papers identified for full-text review. Full-text review and extraction then resulted in a final sample of 13 papers (Table 2), representing four categories of critiques on normative strength and designer agency. These critiques and categories are described more fully in the results section that follows.

## Results

Results from the analysis are presented in Tables 1 and 2. Notable exclusions from the review include sub-approaches outlined earlier (Davis & Nathan, 2015), various iterations of co-design (Sanders & Stappers, 2008) or participatory design (Bannon & Ehn, 2012) which were not explicitly concerned with ethics and values more broadly, and other approaches not primarily oriented to design as a process of practical creation, such as Ratto's critical making (2011), which identifies primarily as a form of design-oriented research, rather than research-oriented design.

**Table 1:** List of E + VID approaches included in review

Name of approach	Description of approach	Normative orientation	Designer agency
Value sensitive design (Friedman et al., 2002)	An approach to the design of technology that accounts for human values through an integrative and iterative tripartite methodology consisting of conceptual, empirical, and technical investigations	Weak normative orientation. Values refer to what a person or a group considers important in life. A list of values of ‘ethical import’ are presented for consideration by the designer	High designer agency. The approach pre-supposes the ability of the designer to identify relevant values, give priority to indirect stakeholders who are strongly affected, and to inscribe them in practice
Design justice (Costanza-Chock, 2018, 2020)	A field of theory and practice concerned with how the design of objects and systems influences the distribution of risks, harms, and benefits among various groups of people	Strong normative orientation. Design justice is concerned with using design to sustain, heal and empower communities while attending to values reproduced in social relations underpinning the design process itself, including how these are constituted within a wider ‘matrix of domination’	Moderate designer agency. The approach emphasizes that designers themselves are part of a broader ‘matrix of domination’, but also mobilizes agentic language that conveys the ability of designers to heal and empower communities
Ethicist as designer (van Wynsberghe & Robbins, 2014)	A series of steps (uncovering relevant values, scrutinizing these values and, working towards the translation of values into technical content) for an ethicist to take in order to fulfill their role as designer on an interdisciplinary team	Weak normative orientation. Ethics ought to be pragmatic, provide utility for the design process, and be informed by an ethicist with knowledge of the relevant issues, rather than an ethical theory or perspective	High designer agency. The approach emphasizes the ability of ethicists embedded in design teams to identify positive values, scrutinize them in light of competing values, and translate them into the technical content of an artifact
Post-colonial computing (Irani et al., 2010)	An alternative sensibility to the process of design and analysis which asserts a series of questions and concerns inspired by the conditions of post-coloniality	Strong normative orientation. The authors take as their starting point a post-colonial discourse centred on questions of power, authority, legitimacy, participation, and cultural intelligibility, particularly in the context of a globalized world	Moderate designer agency. The authors suggest that no design practice takes place outside of the economic conditions that make it possible, but also encourages designers to imagine and create new commitments and spaces for design
Values at Play (Flanagan et al., 2005)	A four-step methodology (values discovery, values-based conflict identification, implementation and prototyping, and values verification) for discovery, analysis, and integration of values in technology design	Moderate normative orientation. The authors promote social change through equity, empowerment, and access to technology, through values identified in the design process	High designer agency. The authors suggest that their methodology for discovery, analysis, and integration of values can help those who struggle to find a balance between their own values, and the values of users, stakeholders, and culture
Values-led participatory design (Iversen et al., 2012)	An approach that considers users’ and stakeholders’ values in the design process through a dialogical process of cultivating the emergence of values, developing them, and grounding them into practice	Moderate normative orientation. Designers enact their appreciative judgement of values by engaging in a dialogical process with intended users	High designer agency. The authors propose viewing design methods and participation as means to achieving engagement with values, refining the emergent values, and then translating them into concrete design ideas
Feminist HCI (Bardzell, 2010)	An exploration of how the design and evaluation of interactive systems might be imbued with the commitments of feminism, articulating six key principles: pluralism, participation, advocacy, ecology, embodiment, and self-disclosure	Strong normative orientation. Takes as its departure feminist standpoint theory, which privileges alternative epistemologies in order to bring about political emancipation	High designer agency. Assumes the ability of the designer to identify and engage with the principles espoused in order to bring about political emancipation
Reflective design (Sengers et al., 2005)	Analysis of the ways in which technologies reflect and perpetuate unconscious cultural assumptions through the design, building, and evaluation of new computing devices that reflect alternative possibilities	Moderate normative orientation. Grounded in critical theory, the approach emphasizes ongoing reflection about technology and its relationship to human life, to inform design decisions that can lead to improved quality of life	Moderate designer agency. Cultural assumptions in design processes should be identified, acknowledged, and challenged by the designer as a means to identifying oversights, and opening new design spaces
Worth-centred design (Cockton, 2006)	Previously called value-centered HCI, six meta-principles and five Ds (Donation, Delivery, Degrading, and Destruction) for designing that motivates people to buy, learn, use or recommend a political, personal, organizational, experiential, or spiritual interactive product	Strong normative orientation. Advocates designers take a focus on “worth”, as manifested in people’s motivations, individually or collectively, to invest time, money, energy or commitment	High designer agency. Encourages designers to attend to users’ motivations, and to use those as a starting point for designing worth
Critical technical practice (Agre, 1997; Boehner et al., 2005)	An approach involving critical reflection on technology production, with special attention to how unconscious assumptions or values are reflected in a field of practice	Moderate normative orientation. Designers are encouraged to engage in critical reflection, thereby uncovering hidden values and assumptions in technology design, and embedding positive values such as critical reflection, emotional experience, and interpretive flexibility	High designer agency. Suggests strategies for altering designers’ work routines, in order to identify values that systems ought to express, and then bringing those values back to the technology design process
Embedded ethicist (Van der Burg, 2009)	An approach focusing on how ethicists who are embedded in a technology design or research project can co-shape new technologies in ethically sound ways	Weak normative orientation. Aspires to broaden the imagination of designers and ethicists beyond technical advances, to the ways in which they change human practices	High designer agency. Emphasizes the ability of the ethicist to enlarge the imaginative scope of a design project
Positive design (Desmet & Pohlmeyer, 2013)	Concerned with how design can contribute to the subjective well-being of individuals and communities, especially: pleasure, personal significance and virtue	Strong normative orientation. Attention is paid to the effects of design on the subjective well-being of individuals and communities, with three main components: pleasure, personal significance and virtue	High designer agency. Each component may necessitate a different approach to design, and it is important that design processes take a holistic approach that capitalizes on the multitude of influencing factors
Design for existential crisis (Light et al., 2017)	How to design for the common good, focusing on human needs for meaning, fulfillment, dignity and decency through four modes of design: being attentive, critical, different, and in it together	Strong normative orientation. Moving beyond critique to using design as principled resistance	High designer agency. An emphasis on the kinds of choices designers (and those commissioning them) make in responding to contemporary crises
Disclosive computer ethics (Brey, 2000)	Concerned with the moral deciphering of embedded values and norms in computer systems, applications and practices. Four key values are proposed: justice, autonomy, democracy and privacy	Moderate normative orientation. Four key values are proposed, however moral theory is thought to present challenges to disclosive analysis: it makes acceptance of a disclosive analysis dependent on the acceptance of a particular moral theory, which may contain pre-conceptions about the technology or practice under scrutiny	High designer agency. It is suggested that disclosive analysis can be a challenging but achievable venture between computer scientists, social scientists and philosophers
Responsible design (Grimpe et al., 2014)	A comparison of responsible research and innovation (RRI) literature to initiatives in human–computer interaction (HCI). Identifies similarities and differences, and outlines challenges associated with realizing an RRI agenda in design	Moderate normative orientation. Advances the four analytic concepts of RRI (reflexivity, responsiveness, inclusion, anticipation) and makes the case for a pragmatic, ‘unromantic’ reinterpretation of RRI for HCI	Moderate designer agency. Emphasizes challenges related to accomplishing an RRI agenda (user and stakeholder perspectives; scope and scale of HCI work; and temporality) and argues for a shared conception of responsibility that demands distributed effort across many parties
Values as hypotheses (JafariNaimi et al., 2015)	No single interpretation of values serves all situations. Values serve as hypotheses by which to examine what the situation is, what the possible courses of action are, and how they might transform the situation	Weak normative orientation. Drawing on the pragmatism of John Dewey, the authors suggest that moral values are neither unchangeable truths, nor local expressions preferences, but a practical matter concerned with the question of action	Moderate designer agency. Design judgments are the outcome of practical, intellectual, and emotional interaction with situations that are indeterminate or puzzling
Value-based engineering for ethics by design (Spiekermann & Winkler, 2020)	An approach to ethical engineering design that draws from software engineering, value sensitive design, design thinking, participatory design, and material ethics of value	Strong normative orientation. Embraces a clear values ontology based on Max Scheler’s material ethics of value. Values are not just a preference or opinion, but are given a priori in any design scenario	Moderate designer agency. Emphasizes the importance of attending to the entirety of a sociotechnical system, and suggests that designers involve corporate leadership and other stakeholders in ‘signing on’ to values priorities and decisions
Virtuous practice design (Reijers & Gordijn, 2019)	An alternative approach to VSD grounded in virtue ethics, expanding the focus from technology design to the technical practices in which artifacts and systems are involved by (1) investigating narratives, (2) reflecting on the practices captured by narratives and (3) prescribing aspects of relevant practices to enhance the cultivation of values	Strong normative orientation. The authors suggest moving from a heuristic of ‘values’ (VSD) to a heuristic of virtues, informed by the tradition of virtue ethics, while also expanding its view beyond the design of individual technologies to technical practices in which they are involved	High designer agency. The authors suggest that narrative accounts of technical practices lead to better understanding of ‘standards of excellence’ and prescriptions for human development, design and regulation

**Table 2:** List of E + VID critiques included in review

Title and Authors	Description	Position on normative strength	Position on agency
*Critique of E* + *VID from a specific normative approach*			
Refining value sensitive design: a (capability-based) procedural ethics approach to technological design for well-being (Cenci & Cawthorne, 2020)	Using a humanitarian cargo drone study as a starting point, the authors argue that VSD’s ethical-democratic import would be enhanced by a procedural ethics stance and deliberative approach inspired by Amartya Sen’s (2004) capability approach	Moderate normative orientation. The authors argue that proceduralism offers significant theoretical, epistemic, and practical-operational insights over substantive ethical theories most commonly employed in VSD, however their chosen theoretical stance does not advance a strong normative or political vision	Moderate designer agency. The authors argue that a procedural approach enhances values pluralism, as well as agency, autonomy, and self-determination of stakeholders. Procedural-deliberative tenets underlying Sen’s account are structurally open to accommodate agents’ diversity and values pluralism, and thus are better able to handle epistemic and moral disagreements
Thinking about design: critical theory of technology and the design process (Feng & Feenberg, 2008)	An analysis of design from three theoretical perspectives: strong intentionality, weak intentionality, and questioning intentionality. The authors describe a viewpoint from critical theory suggesting that culture and history profoundly shape the possibilities of design. The conclusion is that neither the proximate designer nor the immediate design environment is a primary determinant of the nature of the designed product	Strong normative orientation. The authors support a strong moral position aligned to reducing oppression and domination in the world	Low designer agency. The theoretical perspective described is one that outlines the salient and pervasive influences on designer actions, thereby minimizing the agency of the designer in the design process
Capability sensitive design (CSD) for health and wellbeing technologies (Jacobs, 2020)	Merges value sensitive design (VSD) and Martha Nussbaum’s capability theory (2001) to normatively assess technology design with a focus specifically on health and wellbeing	Moderate normative orientation. Nussbaum’s capability theory argues that all people are morally equal and deserve a life worth living, and that every human being should have access to ten central capabilities. However, Nussbaum emphasizes that design requirements should account for local differences, and that the list of ten capabilities is not definite, but open for discussion and revision	High designer agency. CSD presumes the ability of designers to translate abstract capabilities into concrete design requirements by identifying stakeholders, specifying selected capabilities, resolving capabilities conflicts, prototyping the technology, and making adjustments up to the point that the technology finds its ‘ideal’ form
*Summary of critiques of E* + *VID and proposed way forward*			
Next steps for value sensitive design (Borning & Muller, 2012)	A critical review of conventional practices in reporting on VSD activities, proposing four strategies to enhance the rigour and utility of VSD in the future. Strategies relate to (1) ‘tempering’ the claim of universal values, (2) providing stronger context for proposed lists of relevant values, (3) strengthening participants’ voices in reporting on VSD activities, and (4) clarifying the voice of the researcher or author	Weak normative orientation. The authors defend a position in which only those values fundamental to VSD ought to be viewed as necessary, such as pluralism or inclusivity. Additional values would then be included that arise from design participants and the designer, negotiated throughout the design process	High designer agency. The authors imply the capacity of the designer to make decisions about which values to include in the design process and which to exclude
Why value sensitive design needs ethical commitments (Jacobs & Huldtgren, 2018)	A critical perspective on the role of ‘values’ in VSD. The authors argue that VSD practitioners should use ethical theory to complement the VSD process; discuss whether VSD can rely on a set of universal values; and address which ethical theories are best suited to accompanying VSD	Weak normative orientation. The authors argue in favour of a ‘mid-level’ ethical theory such as Beauchamp and Childress’ principalism, or a capability-based approach. They argue that these theories are able to overcome the naturalistic fallacy (the fact that people hold certain values is not sufficient to justify those values), however they do not advance a particular moral theory or normatively strong vision	High designer agency. The authors imply the capacity of the designer to make decisions about which normative theory to apply to their work
*Empirical illustration of challenges in E* + *VID*			
Values and pragmatic action: the challenges of introducing ethical intelligence in technical design communities (Manders-Huits & Zimmer, 2009)	Reports on two attempts to insert ‘ethical intelligence’ into technical design communities. Three key challenges are discussed: (1) confronting competing values; (2) identifying the role of the values advocate; and (3) the justification of a values framework	Weak normative orientation. The authors suggest that a value conscious design project needs to be framed in terms of the normative stance one wishes to take, and that value choices should be based on well-considered ethical judgments, coherent with how designers think the world is best served and structured from a moral perspective	High designer agency. The authors suggest that an important first step in the front-loading of ethics is establishing the role of a values advocate on technical design teams. The role of the advocate is not only to discover and clarify values, but to consciously and deliberately build them into design, even if they conflict with other design objectives
The need for ethical reflection in engineering design: the relevance of type of design and design hierarchy (van de Poel & van Gorp, 2006)	Using four cases, and Vincenti’s (1992) distinction between normal and radical design, the paper explores whether a need for ethical reflection on the part of designers is dependent on the type of design process	Weak normative orientation. The authors suggest that external constraints do not necessarily relieve designers from the need for ethical reflection, but they do not elaborate on the nature of strength of that reflection	High designer agency. The authors imply the capacity of the designer to reflect on and account for the ethically relevant choices they make during the design process
Values as lived experience: evolving value sensitive design in support of value discovery (Le Dantec et al., 2009)	A critical examination of the role of value classifications in VSD, suggesting a more exploratory approach to empirical investigations that treat values as local phenomena, expressed in a local vocabulary	Weak normative orientation. The authors suggest the importance of engaging proactively in value discovery, but do not espouse any particular normative position	High designer agency. The authors imply the capacity of the designer to engage proactively in value discovery
Values levers: building ethics into design (Shilton, 2013)	Report of a field study of a laboratory focused on developing approaches to participatory sensing through mobile phones. The author found that various circumstances of the lab environment and practices of the participants acted as ‘value levers’, which occasioned discussion about participants’ values. These levers led participants to confront which values were being built into the technologies	Weak normative orientation. The analysis is focused on locally derived values as opposed to putting forward an argument for the strength of any particular values framework	Moderate designer agency. The analysis presents design work as a matter of routine designer practices, and clearly outlines the constraints on the design process. The analysis makes room for education as a strategy to create change
Engaging values despite neutrality: challenges and approaches to values reflection during the design of internet infrastructure (Shilton, 2018a)	Report of a field study of the development of a technology infrastructure project, the Named Data Networking Project. Identified three strategies that encouraged values reflection among technology developers: (1) long-term engagement with contexts of use, (2) experiencing self-testing, and (3) encountering socio-technical constraints. The author concludes by proposing that activities that encourage praxis among designers are the ones that will be effective in promoting ethical design	Weak normative orientation. The values that are imaginable to designers are those that are a part of their cultural repertoire. When designers are encouraged to reflect on values being incorporated into design, they come from values already known to the design team. Actors encouraging ethical reflection must navigate these competing values in order to make progress of any kind, and thus the low normative strength position taken in this paper is an empirical observation, not a normative argument	High designer agency. The analysis suggests that by engaging in praxis, designers will become more capable of engaging with values and enacting ethical design
*Reflexivity and Agency in E* + *VID*			
The application of ethics to engineering and the engineer’s moral responsibility: perspectives for a research agenda (Grunwald, 2001)	The author provides a clear and detailed outline of the nature of the normative frameworks that have bearing on designer activities. The author proposes that a more sophisticated understanding of both normativity and individual responsibility is essential for understanding the scope of ethics in design practice, and delineate a set of circumstances in which normative reflection by the designer is necessary	Moderate normative orientation. The author suggests that explicit ethical reflection is not necessary only when an explicit normative framework is brought to bear on the design process. In that the author makes room for explicit normative frameworks in design, there is room for a stronger normative position	Moderate designer agency. The author clearly describes the limitations on designer responsibility, and thereby also designer agency. The responsibility of the designer is described as acknowledging the structures within which they practice, and responding to breaches of important normative agreements embedded within those structures
Reflexivity and value sensitive design (Timmersman & Mittelstadt, 2014)	An argument that the relatively prescriptive and heuristic nature of VSD methodology overlooks the central importance of designer reflexivity in establishing an ethical approach to design. The authors propose that the stronger incorporation of designer reflexivity, and clearer awareness of how the designer’s own values frame and mediate the incorporation of stakeholders’ values, are essential	Weak normative orientation. The authors advocate for an approach in which the designer can more authentically represent the values of the stakeholders, as opposed to incorporating a particular normative position outlined by the designers or design team	High designer agency. Reflexivity is viewed as a capability that can be turned on and off by the designer, reflecting designer agency in whether and how reflexivity is employed
Regulation or responsibility? autonomy, moral imagination, and engineering (Coeckelbergh, 2006)	A discussion of the nature of and contrast between efforts to promote more ethical engineering practice through enhanced external constraints (i.e., regulation) versus promoting greater autonomy. The author proposes a more nuanced understanding of the relationship between the two methods of promoting responsible engineering practice, and outlines the central importance of promoting moral imagination among engineers	Weak normative orientation. The author defers to the moral imagination of engineers. The primary normative position outlined is one focused on minimizing harms associated with disaster	Moderate designer agency. Agency is described as depending on particular structures, including external regulation. Designers are described as having substantial choice within those constraints

We found that normative strength was fairly evenly distributed across categories (weak: 4; moderate: 6; strong: 8). Approaches with a strong normative orientation varied in the values or ethical theories they espoused, but had in common the view that those values or theories ought to substantially influence the design process. Social justice was among the most common (Bardzell, 2010; Brey, 2000; Costanza-Chock, 2018, 2020; Irani et al., 2010), however equity, empowerment and diversity (Bardzell, 2010), happiness (Desmet & Pohlmeyer, 2013), meaning, fulfillment, dignity and decency (Light et al., 2017), worth (Cockton, 2006), and autonomy, democracy and privacy (Brey, 2000) were also advocated. Irani et al. (2010) for example took as their starting point “a discourse centered on the questions of power, authority, legitimacy, participation, and intelligibility in the contexts of [the] cultural encounter” (p. 1).

Approaches considered normatively moderate described processes or principles that ought to be followed during design activities, such as stakeholder involvement and dialogue (Grimpe et al., 2014; Iversen et al., 2012); reflexivity, responsiveness, inclusion, and anticipation (Grimpe et al., 2014); and reflection as an approach to questioning cultural assumptions (Agre, 1997; Boehner et al., 2005; Sengers et al., 2005). However, these normatively moderate approaches did not as clearly articulate goals related to the specific moral outcomes of design in the way observed in normatively strong approaches.

Approaches considered normatively weak sometimes suggested values that the designer might consider (Friedman et al., 2002) but did not suggest which values or normative ethical theories ought to be brought to the process (Van der Burg, 2009; van Wynsberghe & Robbins, 2014). The justification for this approach, well summarized by van Wynsberghe and Robbins (2014), was often the view that “ethics ought to be pragmatic and to provide utility for the design process” (p. 947). Such an approach led to more locally driven approaches to values discovery in the approaches reviewed in our study.

Few approaches explicitly advocated any one particular ethical theory; rather, where normative concerns were most prominent they generally appeared in the form of specific *value* commitments such as a commitment to social justice. In fact, in some cases ethical theory was identified as a limitation. For example, van Wynsberghe and Robbins (2014) note: “rather than using a specific ethical theory to prescribe design changes, ethics in the lab should elucidate morally relevant features of research to force explicit ethical decisions and value tradeoffs by the designer or the engineer.” (p. 950). Brey (2000) suggests that subscribing to any particular ethical theory inevitably requires prior acceptance of that theory based on observable phenomena or empirical presuppositions, and may therefore facilitate conclusions based on preconceptions, where more neutral descriptions may be preferable. Advocating for particular values in place of ethical theory was a strategy to clearly identify the normative content of design while avoiding the often intractable debate associated with comprehensive ethical theories. For example, inspired by Dewey’s pragmatism, JafariNaimi et al. (2015) positioned values simply as hypotheses concerned with questions of action—“What are the conditions which require action, and what is the action which they demand?” (p. 96).

Few frameworks explicitly acknowledged designer agency, but rather were built upon implicit assumptions about the nature of agency in the design process. Views on designer agency fell only into categories of moderate (6 approaches) and high (12 approaches). Approaches which pre-supposed a high degree of designer agency suggested various strategies for engaging with ethics in practice, including new approaches to the designer’s work routine (Agre, 1997), the kinds of choices that designers make (Light et al., 2017), and cooperation or collaboration with other researchers, stakeholders, or designers (Brey, 2000; Friedman et al., 2002; Iversen et al., 2012; van Wynsberghe & Robbins, 2014). Approaches which pre-supposed moderate designer agency focused on the social, cultural, economic, and technical conditions of designers’ priorities and ways of knowing, and how those practices intervened on conceptualizations of system boundaries (Spiekermann & Winkler, 2020), existing cultural practices (Irani et al., 2010), and the ways in which unconscious values were embedded in design (Sengers et al., 2005). Only responsible design (Grimpe et al., 2014) and value-based engineering by design (Spiekermann & Winkler, 2020) explicitly acknowledged the temporal aspects of design, noting its relevance to designer reflexivity, organizational and institutional practices, and designer responsiveness to stakeholder needs and contexts of use. Acknowledging the intersection of these influences on design is characteristic of approaches with a moderate view of designer agency. No frameworks in our first review were found to espouse a view of low designer agency, which would strongly emphasize the influence of the institutional, cultural, or political influences on designers.

Many approaches emphasized the importance of designer reflexivity or reflection in attending to the moral or ethical dimensions of design. Sengers et al. (2005) defines reflection on technology and its relationship to human life as “bringing unconscious aspects of experience to conscious awareness, thereby making them available for conscious choice” (p. 50). For Sengers, this must not just occur in the design process, but must be a core outcome for technology *users* too. Critical technical practice suggests that the primary task of the designer is to engage in the reflexive work of critique (Agre, 1997; Boehner et al., 2005). Grimpe et al. (2014) point to calls for both designer and institutional reflexivity in the face of challenges presented by the design process (noting in particular temporality, perspective, and scope) and suggests that reflexivity in its own right is not a virtue, nor is it a fixed one-time event, but rather, is a relational exercise that must be ongoing.

### Critiques of Literature on Ethics and Values in Design

The results of our second search are presented in Table 2. We included 13 articles in the final sample of our second search, selected for their strong resonance with the objectives of this second search and their representation of four distinct categories of critique levied against E + VID literature. We grouped the articles included in the second search into those that (a) critiqued E + VID literature from a particular normative position (Cenci & Cawthorne, 2020; Feng & Feenberg, 2008; Jacobs, 2020), (b) summarized existing critiques of E + VID in the literature and proposed a particular way forward (Borning & Muller, 2012; Jacobs & Huldtgren, 2018), (c) reported findings from empirical research illustrating the challenges of E + VID (Le Dantec et al., 2009; Manders-Huits & Zimmer, 2009; Shilton, 2013, 2018a; van de Poel & van Gorp, 2006) and (d) elaborated the significance of reflexivity specifically as it relates to designer agency (Coeckelbergh, 2006; Grunwald, 2001; Timmermans, 2017).

We identified three articles that critiqued E + VID literature from a particular normative position. Cenci and Cawthorne (2020) proposed a capabilities-based approach to E + VID informed by the work of Amartya Sen (2004), and argued that the ethical approaches in Value Sensitive Design in particular are not capable of addressing the more complex normative issues arising from new technologies. Feng and Feenberg (2008) drew on Andrew Feenberg’s critical theory of technology to outline how popular approaches to E + VID lack a clear normative vision and misunderstand the links between human agency and technology. Jacobs (2020) drew on Martha Nussbaum's (2001) capability theory to normatively assess technology design, with a focus on health and well-being. We categorized Cenci and Cawthorne (2020) as advocating a view of moderate designer agency and moderate normative strength, Feng and Feenberg (2008) advocating a view of low designer agency, and high normative strength, and Jacobs (2020) advocating a view of high designer agency, and moderate normative strength.

We identified two articles that reviewed E + VID literature in order to identify existing critiques, and propose ways forward for the field (Borning & Muller, 2012; Jacobs & Huldtgren, 2018). Both of these articles engaged with the status of values in E + VID, critiquing the possibility of articulating universal values and outlining theoretical positions to strengthen the empirical accuracy of E + VID approaches. We classified both of these articles as situated in a view of high designer agency and low normative strength.

We identified five articles for inclusion in our review that focused specifically on documenting challenges with the practice of E + VID through empirical research. Two of the included papers assessed the mechanisms through which values are built into the design of technologies (Manders-Huits & Zimmer, 2009; Shilton, 2013) and three addressed the role of ethical reflection in shaping the categories of values that were viewed as the foundation for the design process (Le Dantec et al., 2009; Shilton, 2018a; van de Poel & van Gorp, 2006). All but one of the included articles was embedded in assumptions of high designer agency (Shilton, 2013 espoused moderate designer agency), and all included articles espoused views of low normative strength.

We found three articles for inclusion in the final category, comprised of those focused specifically on the role of reflexivity and its implications for agency in design activities. Two of the included articles proposed a greater emphasis on designer reflexivity as a way to enhance the agentic capabilities of the designer in considering and incorporating ethical aims into the design process (Grunwald, 2001; Timmermans, 2017). The final article proposed further developing the concept of moral imagination among designers as a central avenue for future work (Coeckelbergh, 2006). The emphasis on moral imagination was put forward in the context of the clearly described tension between (a) enhancing constraints on design (such as regulation), and (b) allowing greater designer autonomy as competing approaches to promoting more ethical outcomes from the design of new technologies. Articles on reflexivity in design included those that described normative strength as moderate (Grunwald, 2001) and low (Coeckelbergh, 2006; Timmermans, 2017), and those that described designer agency as high (Timmermans, 2017) or moderate (Coeckelbergh, 2006; Grunwald, 2001).

## Discussion

We completed a structured review of E + VID approaches and classified them according to their positions on designer agency and the normative strength of ethics and values brought to the design process. We then reviewed literature that provides critiques of E + VID approaches, categorized them according to the nature of their contribution, and outlined the assumptions they espouse regarding designer agency and normative strength. We found that none of the original approaches arising from our first search represented a view characteristic of “low” designer agency, and that the approaches we summarized were spread across the spectrum of views regarding normative strength. Only one article critiquing E + VID included a view of low designer agency (Feng & Feenberg, 2008), and the critiques were also spread across the spectrum with respect to normative strength. These findings suggest that E + VID literature is generally characterized by a set of assumptions that locate responsibility for ethical design decisions primarily with individual designers, positioning the designer as the source of decision-making power about whether and how a particular normative position will guide the design process. We suggest these assumptions are deeply linked with the normative potential of E + VID.

In this discussion section we first provide a relatively detailed description of two competing approaches to understanding human agency that are prominent in contemporary social sciences: a relational view of agency, and an actor network view of agency. We have selected these two approaches to understanding human agency not because we advocate for their use in conceptualizing design activity per se, but simply to illustrate two sophisticated and popular ways of understanding human agency to clarify the dimensions of the theoretical challenge at hand. We outline the implications of these two ways of understanding agency for conceptualizing the work of the designer, and explicitly discuss their implications for the normative status of design activity. We then conclude by proposing directions for future work that can help to push forward the theoretical work required to strengthen the foundation of E + VID.

### A Relational View on Designer Agency

Emirbayer and Mische (1998) provided a comprehensive multi-theoretical review of the concept of human agency that ultimately built upon the influential works of Bourdieu (1990) and Giddens (1979) in viewing agency and structure as co-constitutive. This is commonly known as the “paradox of embedded agency”—actors can exert influence on systems, but systems themselves are also seen as constructing agency (Garud et al., 2007; Seo & Creed, 2002). This phenomenon has been addressed by a wide range of social theorists, and the relational view of agency can be seen as closely related to the body of work on practice theory as an explanation of human action (Hui et al., 2016; Nicolini, 2012; Ortner, 2006). In their review, Emirbayer and Mische (1998) set out to analytically disaggregate agency from structure, defining agency as:

The temporally constructed engagement by actors of different structural environments - the temporal-relational contexts of action - which, through the interplay of habit, imagination, and judgment, both reproduces and transforms those structures in interactive response to the problems posed by changing historical situations (Emirbayer & Mische, 1998, p. 970).

This definition encompasses Emirbayer & Mische’s three elements of human agency: iteration, projectivity, and practical evaluation. We briefly review each major constituent below.

The *iterational* element is perhaps best understood as a “past” orientation, whereby patterns, habits, or norms are reproduced through actors’ routines, giving stability to institutions and sustaining agentic identity over time. Through these schemas for example, designers may recall, select, and apply tacit knowledge acquired through past experiences. The *projective* element may be considered a reflective “future” orientation, were actors hypothesize potential future trajectories and actions in response to challenges or uncertainty. They may distance themselves from constraining schemas. The specific, culturally embedded ways in which these projections occur affect the extent to which designers experience freedom within existing structures. The *practical-evaluative element* of agency is oriented to the present, whereby actors make practical judgements about alternative possibilities in response to emerging demands, dilemmas, or ambiguities in ever-changing situations. Actors with strong practical-evaluative skills are supposedly better able to act as mediators who contextualize social experience. Importantly, Emirbayer and Mische (1998) emphasize that all three of these elements can be found to varying degrees in any situation of action, and that they may sometimes be in conflict. As actors alter or shift their agentic orientation through intersubjectivity, social interaction, and communication, they increase or decrease their capacity for transformative impact.

The view espoused by Emirbayer and Mische (1998) makes plenty of room for individual creativity and spontaneous action, represented especially by the practical-evaluative element of agency but also by the statement that human action arises as a result of the confluence of all three. This view on agency is theoretically sophisticated, and has been used to critique naïve ethical systems that rely on personal responsibility and a simplistic understanding of moral agency (Skalko & Cherry, 2016). The relational view on agency likely forms an implicit assumption of many of the E + VID approaches included in our review, as it is capable of acknowledging the influence of forces external to the proximate designer without eclipsing the designer’s capacity for creativity and agency (Feng & Feenberg, 2008). Indeed, the articles grouped into the “reflexivity and agency in E + VID” category of our second search all resonate with the approach to agency articulated here.

Certain articles identified in our second search departed from the relational view of agency described here in two particular ways. First, Feng and Feenberg (2008) explicitly engaged with the influences on human agency found in power-laden structural environments, and used this position as a critique of the assumption that designers have meaningful control over design decisions. This critique is the clearest and most direct affront to a relational approach included in our review, as it would contend that all three dimensions of agency outlined by Emirbayer and Mische (1998) are always already shot through with power relations that make them what they are. Second, the empirical studies that drew on theory from science and technology studies identified several instances of nuanced material and environmental influences on design processes that are generated separate and apart from the individuals on the design team (e.g., Shilton, 2018a, b). In so doing, they shift the emphasis toward non-human causes of design decisions.

The contrast between these critiques and the original approaches included in our structured review illustrate one of the notable findings of our review: *That even those E* + *VID approaches that embraced the most critical view of the world, such as design justice and feminist HCI, did not embrace a “low agency” view of the designer.* This point is of interest because these theoretical systems are those that most strongly acknowledge the profound impact of power, manifested through politics, gender, the economy, and other phenomena, in the constitution of human action and the lived world (Bardzell, 2010; Costanza-Chock, 2018, 2020). Although these E + VID approaches acknowledge the obligation to articulate a clear normative vision about the purpose of design based on the potential impact of designed objects once they are deployed in the world, the same consideration has not been made for the heavy influence of the world on the thoughts and actions of the designer. The proposed principles of the Design Justice Network by Costanza-Chock (2018, 2020), for example, mobilizes agentic language such as, “We use design to sustain, heal and empower…” and, “We center the voices of those who are directly impacted…” (p. 11). Spiekermann and Winkler (2020) suggest that “the engineering organization should embrace a culture of openness, transparency and genuine care for doing good” (p. 6) and that designers “should have an acknowledged or a managed relationship” with broader socio-technical “system of systems” partners. Such language conveys an agentic designer making reflective choices to engage in the practice of design in particular ways.

Our observation of the absence of low designer agency approaches is not in itself a critique of this literature (indeed we view many of these approaches very favourably), but is simply an illustration that E + VID might have reason to avoid theoretical approaches that minimize the role of human agency in producing action and change. However, before we address this point in greater depth, we turn to a second approach to understanding human agency in Actor Network Theory.

### Actor Network Theory and Designer Agency

Actor Network Theory (ANT) has become a popular theoretical ‘toolkit’ in the social sciences (Asdal & Moser, 2012), representing an approach to analyzing action and change that departs from many of the philosophical foundations otherwise taken for granted in the field (Latour, 2005). Despite acknowledged challenges with ANT as a theory in itself (Latour, 1999), the alternative questions about the world posed by this approach have generated a great deal of interest in the study of science, technology and “social” concerns more generally (Gad & Jensen, 2010). A fundamental move at the root of ANT and its allied theoretical approaches is to shift from using accepted categories such as ‘the social’ or ‘human agency’ to explain phenomena about the world, and instead to treat those very perceived realities as the phenomena to be explained in the first place. For Latour (2005), this means embracing uncertainty in order to benefit from the inquisitive stance of the social sciences: “The interesting question at this point is not to decide who is acting and how but to shift from a certainty about action to an uncertainty about action… As soon as we open again the full range of uncertainties about agencies, we recover the powerful intuition that lies at the origin of the social sciences” (Latour, 2005, p. 60).

ANT famously adopts an orientation to explaining phenomena in the world by describing the various elements participating in the constitution of those phenomena; in the language of ANT, these are the “actants” that come together to form the associations or actor-networks that we recognize as a particular reality (Latour, 1999, 2005). In this approach, the act of designing is as much caused by tables, chairs, whiteboards, sticky notes, coffee, a room of a particular size/shape, and multi-colored pens as it is by the humans participating in a design exercise. Each of those actants brings something new to the actor-network that makes up the practice of design in that instance. However, the actor-network does not stop there. It also includes the buzzing phone displaying emails from the manager outlining project deadlines, the push notifications about the latest political event that raises the ire of the designer, and the photo on the laptop background showing the next vacation travel destination. These objects that have presence in the design room represent realities that extend beyond the design team itself, yet are present as the design process unfolds nonetheless.

This view takes an approach that levels human agency way down to size, questioning the assumptions about the creative freedom and talent of the proximate designer. An ANT-inspired approach to human agency suggests that if you want to understand human agency (or creative freedom or talent or any other characteristic of the designer), you need to look to all of the other objects and realities (i.e., actants) that make it what it is. This would require looking to the wider range of influences on the designer, not only acknowledging their impact on the design process, but also *making effort to intervene upon them as part of the practice of design*. This is in our view the most important point regarding human agency that appears to have been overlooked by the E + VID approaches included in our review: by neglecting to acknowledge the profound impact of the many influences on the agency of the designer, these approaches neglect to identify those influences as important targets of intervention by the designer.

#### Reflections on Designer Agency

The approaches included in our review were only rarely critical of conventional understandings of human agency as it relates to design activities. However, ultimately this point is understandable. If the designer is not viewed as an agent who can act freely in the world, then who or what would enact the outputs of an approach to design advocating for a stronger moral orientation? Design is a field based on the assumption that the designer can act in free and creative ways; it is a notable challenge to acknowledge the salience of influences on designer agency (and thereby embrace a “low” designer agency view) while simultaneously creating recommendations for designers to purposefully act in different, more ethical ways.

One obvious reason that the E + VID approaches included in our review do not embrace a low designer agency perspective is that doing so leads to the theoretical challenge of making space for creativity and intervention while simultaneously acknowledging the profound impact of external influences on designer agency. Although this is a legitimate theoretical challenge, we suggest that it is precisely the sort of theoretical inquiry with which E + VID literature ought to engage. Doing so may help to clarify points of intervention that are necessary to align normatively strong positions held by designers with broader influences on the design process, making E + VID more conceptually and practically feasible.

Ultimately, we suggest that further engagement with interdisciplinary theory on human agency will accomplish two goals. First, it will enhance the clarity of the role of the designer in enacting more ethically sound approaches to design. Greater clarity about the nature of creativity and decision-making with respect to design choices will provide a foundation for even more sophisticated understandings of where to direct our attention in support of ethical design. Second, and relatedly, it will support the identification of points of intervention that enable designers to engage in normatively stronger design work. When we understand the nature of the influences on designers and design work, it becomes possible to shape them in ways that support particular aims.

To better illustrate our point, we might imagine points of intervention at three different levels. At a broader societal level, one might act on consumer expectations regarding data as an influence on the design process. Regional differences in data protection and privacy illustrate the values-laden nature of the topic, but only rarely is it explicitly discussed as centrally relevant to the work of technology designers, or to values brought to the design process. At the organizational level, one might investigate incentives that shape managerial decisions. The impacts of corporate surveillance, censorship, and discipline on socially conscious designers, for example, underscores the importance of attending to both the normative dimensions of design, as well as influences on the agential capacities of designers that shape their ability to realize a positive vision for their work. At the individual level, one might examine how these and other influences come together to shape the expectations of designers regarding the work they do. While we have outlined two such examples here, these influences are innumerable and context-dependent, and will no doubt provide a foundation for considerable research in years to come.

We acknowledge that the logic we espouse here, that enhanced engagement with the theoretical challenge of designer agency will enhance our ability to act in morally superior ways, makes the very assumption about agency we are aiming to critique. By proposing that the E + VID community ought to engage with a certain set of concepts, we are implying that members of that community can make free decisions to read the body of work we propose and to conceptualize E + VID in potentially new ways. However, we propose this direction on the observation that human thoughts and decisions do indeed impact the development of bodies of work and the evolution of fields of practice. We can bracket for the time being the exact mechanism by which reading an academic paper might influence the thoughts and actions of design scholars, because we know it has potential to do so. Perhaps this same insight is at the root of the “high agency” view of designers in the E + VID literature, but regardless, we maintain that further attention to this point will only enhance the ability of E + VID approaches to promote the normative aims of design ethics. This point also reiterates the importance of attending to this theoretical dilemma in sustained future work.

#### Reflections on Normative Strength

The approaches included in our review reflected the entire range of the spectrum of normative strength we set out in our classification system. Having categorized approaches based on how strongly they advocated for a particular method of considering ethics and values during the design process, the common feature of those classified as normatively strong became more evident: They each articulate a vision (albeit with various degrees of clarity) of a better future that is sought after through the design practices they promote. Where approaches classified as normatively moderate outlined particular processes for identifying ethics and values, and those classified as normatively weak outlined options as opposed to suggestions, the normatively strong approaches provided descriptions of the world that represent a morally better future that ought to be pursued. In this section we provide an example and more detailed description of a normatively strong approach, and comment on the role of normative strength in building the field of E + VID.

One example of a normatively strong approach included in our review is postcolonial computing (Irani et al., 2010). This approach is motivated by the normative and theoretical concerns of postcolonial studies, which seeks to identify the harms associated with the flow of economic systems, technological products, and ideas about society from global powers to less geopolitically powerful nations and cultures (Irani et al., 2010). By stating that postcolonial computing is “a project of understanding how all design research and practice is culturally located and power laden,” (p. 1312), Irani et al. emphasize the ways in which design inadvertently participates in the ongoing work of colonialism. The cases they present in their paper included in our review outline the harms caused by the consequences of design decisions driven by corporate capitalism for developing nations, thereby advocating for design practices that are driven more by local needs and cultures than by the demands of global capitalism. This vision for design as one that is locally relevant, culturally respectful, and resists the negative consequences of global capitalism, represents a morally coherent goal for design theory and practice. The vision for the future underlying this vision of design is one that promotes the flourishing of local knowledges and cultures, and mitigates against the damages caused by corporate capitalism.

Ultimately, we suggest that the challenges associated with advancing such a strong normative vision for the impact of design in the world confronts the same challenges outlined in the previous section on designer agency. These challenges relate to the fact that technology design largely takes place within contexts that are oriented toward building technologies that can be deeply embedded in various segments of the economy and eventually become a perceived necessity among consumers. When these expectations frame the practice of much design that takes place in the world, such as in corporate design contexts, how can a normatively strong vision for the future meaningfully characterize design practice? Under the conditions outlined here, a normatively strong orientation to E + VID appears as wishful thinking.

At this point, the observations we have made about normative strength intersect with our observations about beliefs regarding designer agency. Just as a normatively strong orientation to E + VID appears as wishful thinking, a high designer agency approach appears as conceptually flawed. When the corporate expectations of managers responding to global markets have such profound influence on design decisions, how can the designer exercise agency to mobilize one set of normative goals over another? This question opens up crucial lines of research for design scholarship. For example, assuming a degree of agency that enables the design community to intervene in an effort to enable more ethically sound design, what might be the most appropriate points of intervention? As previously outlined, the practices of managers, fundraising strategies, and expectations of consumers are all potential sites of intervention that might evolve in ways to invite more normatively strong approaches to design. We suggest that this line of thinking ought to represent a core preoccupation in the E + VID literature in the years to come, ideally leading to new avenues for the pursuit of E + VID that build upon strategies to enhance designer agency, and engage a broader range of stakeholders in considering and pursuing stronger statements of a morally positive future.

## Future Directions and Conclusion

This structured review represents a starting point for attending to what we believe to be two centrally important concepts for E + VID: designer agency, and normative strength. This bears important implications for the practice of E + VID, where unacknowledged influences on designer agency may inhibit the realization of a more equitable, sustainable, just, or otherwise ‘good’ society. In doing so, the review underscores the value of sustained inquiry into related concepts that have long preoccupied scholars of engineering and design, such as design frames (Dorst, 2011), design requirements (Van de Poel, 2001; van Gorp, 2007), and system boundaries (Ottens, 2009). Specifically, it offers new tools with which to think about possibilities for designer agency that are often assumed by those concepts, particularly when acknowledging the broader range of influences acting on the agency of designers.

We acknowledge that our findings raise many questions about how designers can practically engage with the key messages of our work. Although a comprehensive answer to these questions are beyond the scope of this paper, we outline three directions for future research in design scholarship that will build a foundation for future, more practically-oriented approaches that address and build upon these insights. First, with respect to emerging digital technologies, where considerable design work is undertaken by large technology companies, these findings highlight the importance of investigation into how to intervene upon constraints to designer agency, as well as how to expand it through the formulation of new design strategies. These strategies might be aimed at corporate management, regulators, or others implicated in the design and development ecosystem. Second, E + VID would also benefit from research into the feasibility of any such interventions and strategies, including whether they ought to be comprised of new design methods, practices, or sites, or other formulations tailored to local contexts. Finally, future work should also examine strategies to enhance designers’ understanding of what constitutes a positive moral vision that might inform a normatively strong orientation to design in the context of digital capitalism. If designers are to invest their time and energy in the difficult work of creating more meaningful spaces for designer agency, such work must be situated in a clear and more compelling vision about a morally positive future. Answers to these questions, we argue, constitute a new and promising direction for E + VID, and will demand considerable attention in years to come.
